# Real Time Pulse Chase (RTPC) In-Cell NMR Spectroscopy Reveals Critical Metabolites in Subminute Metabolism of Undifferentiated and Differentiated Human Neuronal Cells

**DOI:** 10.1021/acs.analchem.5c07848

**Published:** 2026-06-18

**Authors:** Spoorthy Sandras, Nicholas Sciolino, Sergey Reverdatto, David S. Burz, Jayanti Pande, Ann Marie Schmidt, Ravichandran Ramasamy, Alexander Shekhtman

**Affiliations:** † Department of Chemistry, 1084 University at Albany, Albany, New York 12222, United States; ‡ Diabetes Research Program, Department of Medicine, New York University Grossman School of Medicine, New York, New York 10016, United States

## Abstract

Neurons undergo extensive metabolic reprogramming during differentiation; this reprogramming leads to specific changes in the kinetics and concentrations of metabolites. We developed an in-cell NMR-based method that monitors this metabolic transformation with subminute time resolution. Undifferentiated SH-SY5Y human neuronal precursor cells were encapsulated into alginate gel beads and differentiated inside the gel. Real time pulse chase (RTPC) in-cell NMR was used to measure relative steady state concentrations of glycolysis and TCA cycle metabolites and the kinetics of metabolite production and clearance in differentiated and undifferentiated cells. Neuronal differentiation slowed glycolysis, increased TCA cycle activity and glutamate production. Fructose 1,6 bisphosphate and glutathione were identified as major biomarkers of undifferentiated and differentiated cells, respectively. The results demonstrate that RTPC-NMR analysis of neuronal cells is an effective method for studying changes in metabolite profiles induced by stress and drug-induced stimuli.

## Graphical abstract

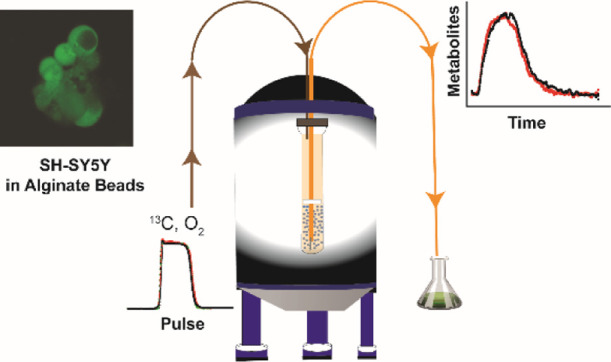

## Introduction

Metabolism plays a critical role in the physiology and pathology of neurons.^1,2^ Neurons primarily use glucose as a source of energy, which is almost entirely sequentially oxidized through glycolysis, the tricarboxylic (TCA) cycle and oxidative phosphorylation (OXPHOS). Although dramatic changes in the activity of glycolysis and the TCA cycle take place during differentiation,^3,4^ relatively few reports have addressed changes in metabolism occurring during this process. To investigate the rates of metabolic changes, a model of terminal differentiation of SH-SY5Y cells was employed. SH-SY5Y cells are a cloned adrenergic subline of SK-N-SH human neuroblastoma cells derived from a metastatic bone tumor that have been extensively used to study neuronal cell biology.^5–8^

Bioreactor NMR spectroscopy has been used to detect real time changes in metabolism of mammalian cell lines by using ^13^C-labeled metabolites^9–12^ or hyperpolarized ^13^C pyruvate and ^13^C-alanine.^13–15^ The time resolution of the studies with ^13^C-metabolites was limited to tens of minutes so that it was impossible to resolve fast kinetics of glycolysis that usually takes place within minutes.^16^ The NMR experiments with hyperpolarized ^13^C-pyruvate and ^13^C-alanine are highly sensitive but limited to short-term studies due to the rapid decay of hyperpolarization. Furthermore, the use of pyruvate and alanine restricts metabolic studies to postglycolytic pathways. Recently developed real time pulse-chase in-cell NMR spectroscopy, RTPC-NMR,^17,18^ provides a unique platform to monitor the relative concentrations of metabolites and quantify the rates at which these concentrations change as live cells undergo various cellular stresses. RTPC-NMR measures changes in the concentrations of metabolites in cells packaged into alginate beads inside a bioreactor as the cells are exposed to ^13^C-labeled glucose in the growth medium. Rates of incorporation and clearance of label are monitored by isotope-selective in-cell NMR experiments^19–22^ collected at 47 s intervals.^17,18^ This technique was used to study changes in the metabolomic rates of HEK293 cells exposed to a model mRNA vaccine.^17^ To date, this technique has only been applied to robust cell lines such as HEK293 or HeLa.

In this study, RTPC-NMR was modified to monitor metabolic changes during the differentiation of human SH-SY5Y cells into dopaminergic neurons. Profound changes in both glycolysis and TCA cycle intermediates were observed upon differentiation. Specifically, a significant decrease in differentiated cells in the glycolytic metabolite fructose-1,6-bisphosphate, FBP, which accelerates glycolysis by acting as a feed-forward activator of pyruvate kinase, and, consistent with previous studies, an increase in reduced glutathione were observed.^23^

## Materials and Methods

### Cell Culture

Human SH-SY5Y neuronal cells were obtained from the American type culture collection (ATCC). No mycoplasma contamination was detected and cell authentication was confirmed using STR profiling. Cells were seeded at 1 × 10^6^ cells per 10 cm plate (Corning) in 20 mL of growth medium, a 1:1 mixture of Dulbecco’s Modified Eagle Medium, DMEM and GlutaMAX supplement containing 4.5 g/L of d-glucose and 110 mg/L of sodium pyruvate (Gibco), and Ham F-12 Nutrient Mix (Gibco), supplemented with 10% fetal bovine serum (Gibco), FBS, and 1% Penicillin–Streptomycin (Sigma-Aldrich) (Pen-Strep), at 37 °C for 48 h (h) in 5% CO_2_ until 80% confluence was achieved. The growth medium was replaced every 2 days after washing the cells with phosphate buffered saline (PBS). Cells were harvested using 0.5% trypsin–EDTA for 5 min at 37 °C, centrifuged at 5 RCF for 20 min at 24 °C, counted using a hemocytometer and resuspended in 20 mL of growth medium at a density of ∼4 × 10^6^ cells/mL for repassage in 15 cm plates. After 4–5 passages, 12 plates yielded a total of cell ∼1 × 10^8^ cells. From this point, the cells were subjected to discrete conditions depending on whether they were to be differentiated or left undifferentiated and whether they were to be imaged, assayed for dopamine or cast into beads and loaded into the bioreactor for in-cell RTPC-NMR spectroscopy.

### Cell Differentiation on Plates

Cells were differentiated in 10 cm culture plates for imaging and dopamine detection assays. The procedure used for differentiation of SH-SY5Y cells was an abbreviated form of that previously reported.^5,24^ Cell culture plates from the final passage were treated with 10 μM retinoic acid (RA) from a 10 mM stock in 1% DMSO, in growth medium at 37 °C and 95% air/5% CO_2_ for 48 h followed by 80 nM tetradecanoyl phorbol acetate, TPA, from a 160 μM DMSO stock solution, for an additional 48 h. The growth medium was changed every 12 h. Undifferentiated and plate-differentiated cells were used for direct imaging.

### Cell Differentiation in Beads

Cells encapsulated in alginate beads were differentiated in 10 cm plates containing growth medium supplemented with nontoxic concentration of 100 μg/mL of kanamycin,^25^ Kan, from a 1000× stock following the same protocol as above. Kanamycin was added only to the alginate-encapsulated samples because the additional handling required for encapsulation introduced a greater risk of contamination. Although the gel preparation and filtration steps were carried out under sterile conditions, bacterial growth was occasionally observed in the encapsulated system.

### Cell Casting into Beads

Undifferentiated cells were cast into alginate beads for imaging, dopamine detection assays and differentiation. The packaging protocol used was previously described^17,26^ with some modifications. To minimize contamination, bead casting was performed in a sterile incubator. Approximately 1 × 10^8^ cells (12 plates) from the final passage were harvested as described above. The cell pellet was resuspended 1:1 (v/v) in 2% alginate (Sigma-Aldrich) in growth medium lacking FBS. To provide a matrix for cell adhesion, bovine Corning collagen I (Sigma-Aldrich) was added from a 3 mg/mL stock solution in PBS to a final concentration of 0.25 mg/mL. The suspension was loaded into a 3 mL syringe fitted with a Luer-Lok tip mounted on a syringe pump (New Era Pump Systems NE-300) and connected to a blunt 21-gauge stainless steel needle with a 40 mm length of Tygon tubing (I.D. 0.79 mm). The needle was oriented at a 45–60° angle and the cell-alginate-collagen suspension was injected into the tip of an atomizer that consisted of a vertically oriented 5 mL serological pipet. Compressed air was filtered by passing through a sealed sterile flask containing sterile deionized water and sterile polyester fiber polyfill (Fairfield). The atomized mixture was uniformly dispersed into a beaker containing 40 mL of 150 mM calcium chloride, CaCl_2_, allowing the Ca^2+^ to polymerize the alginate and encapsulate the cells within uniformly sized beads. The atomizer pressure and cell suspension flow rate were optimized to 5 pounds/square inch (psi) and 300 μL/min to yield beads of uniform shape and size (Figure S1). Excess CaCl_2_ solution was decanted, and the encapsulated beads were transferred to a 10 cm culture plate for further analysis or prepared for RTPC-NMR.

### Calcein Staining and Fluorescence Imaging of Alginate Beads

The cell-permeable hydrophobic stain Calcein AM (Invitrogen) was used in accordance with the manufacturer’s guidelines to investigate cell viability and the presence of dendritic and neurite processes in SK-SY5Y cells. Briefly, cell beads were placed in 15 mL centrifuge tubes (Corning) and suspended in 2 mL of PBS. Ten μL of 1 mg/mL Calcein-AM in DMSO was added and the beads were incubated at RT for 30 min, washed twice with PBS and resuspended in 200–400 μL of PBS. The suspension was transferred to a standard glass microscope slide (VWR) to which a hollow square stack of parafilm was adhered. This created a 400 μL gap between the slide and coverslip forming an enclosed area for the solution and a tight seal that prevented evaporation. Beads were collected immediately after casting and after differentiation. The differentiation timeline proceeded 96 h prior to a second casting of undifferentiated cells, so that both conditions could be stained and imaged at the same time for continuity. Images were taken with a Zeiss LSM 980 using excitation and emission wavelengths of 494 and 514 nm, respectively.

### Dopamine Detection Assays

Undifferentiated and differentiated cell lysates were prepared for analysis of dopamine content. Cells were seeded at a density of 8 × 10^6^ cells/mL and the growth medium was aspirated when the cell culture reached optimal confluency. The cells were harvested by rapid freezing: 4–5 mL of liquid nitrogen were added to the cell culture plate and the frozen cells mechanically detached using a sterile cell scraper and transferred to a 1.5 mL Eppendorf tube. PBS was added to a final volume of 500 μL. The lysate was sonicated at 20% amplitude to facilitate cell disruption and ensure thorough lysis. To prepare cell lysates from alginate beads, the growth medium was aspirated, and the beads were transferred to a 1.5 mL Eppendorf tube using a Pasteur pipet. Four to five mL of liquid nitrogen were added to the cells, followed by sonication. The final volume was adjusted to 500 μL using PBS.

Dopamine was assayed at room temperature (RT) using a Dopamine ELISA kit (Abnova). The competitive ELISA was performed in accordance with the manufacturer’s guidelines. Briefly, 500 μL of undifferentiated and differentiated cell lysates from plate cultures and from beads were added to each well of a 96-well plate along with the appropriate standards and controls. Dopamine was captured on boronate affinity plates extracted, acylated and enzymatically treated with catechol-*O*-methyltransferase and coenzyme *S*-adenosylmethionine. In the ELISA procedure, a rabbit antidopamine primary antibody and secondary antirabbit immunoglobulin-peroxidase conjugate were used in conjunction with a chromogenic substrate containing 3,3′,5,5′-tetramethylbenzidine to generate a signal at 450 nm.

To analyze the data, a standard curve of absorbance versus log concentration, was generated using the standards provided. The standard concentrations were multiplied by a correction factor and the absorbance values from both standards and samples were normalized. The standard curve was fit to *y* = *ax* ^2^ + *bx* + *c* to resolve dopamine concentrations. The results were converted from nmol/mg to pmol/mg protein by multiplying by 1000. Each measurement was performed multiple times to ensure accuracy, and results were reported with standard deviations.

### Bioreactor Setup

The bioreactor setup was used as previously described^17,18^ with some modifications. Approximately 600 μL of beads containing undifferentiated or differentiated cells were loaded into the bioreactor and equilibrated with Krebs-Henseleit (KH) medium, which consists of KH buffer salts (25 mM NaHCO_3_, pH 7.2, 118 mM NaCl, 4.7 mM KCl, 2.5 mM CaCl_2_, 1.2 mM MgCl_2_ and 1 mM NaH_2_PO_4_) supplemented with 5 mM ^12^C-glucose, 0.4 mM palmitate, 0.4 mM BSA and 70 mU/L of insulin to establish a metabolic steady-state prior to the introduction of uniformly labeled glucose, [U–^13^C]-glucose, in KH medium. Beads containing differentiated cells included 100 μg/mL Kan. The flow of medium, prewarmed to 46 °C in a Bransonic CPX2800H ultrasonic cleaner (EDP: CPX-952-218R), was controlled by a programmable peristaltic pump and oxygenated with 95% O_2_/5% CO_2_ to provide sufficient amount of oxygen in encapsulated cells.^27^ A D-150 Membrane Oxygenator (Harvard Apparatus; Item no. 73-3757), which operates on a principle similar to dialysis using hollow polysulfone fiber membranes, was used to introduce the gas mixture to the medium. An inlet gas pressure of 14.8 psi was maintained with a PPR2-N02BG-2 regulator (PneumaticPlus) and the oxygenated medium was delivered into the NMR tube to maintain consistent concentrations of O_2_ and CO_2_. The 95% O_2_/5% CO_2_ gas mixture was used to maintain an adequate level of dissolved oxygen in the perfused medium. Because oxygen transfer is limited by the membrane oxygenator, tubing and encapsulated bead system, a high oxygen partial pressure was required to achieve a medium pO_2_ of approximately 120–150 mmHg, which is in the range commonly associated with standard cell culture normoxia. A drip irrigation stem with a microporous diffuser tip inside the NMR tube ensured proper nutrient flow without disrupting the beads.^17,18^ A Luer-lock latex septum (Qosina) was attached in-line after the Rheodyne 7125 injector to provide sampling access, to allow for oxygen testing and to remove any bubbles that may evolve before they reach the diffuser. To prevent temperature reduction from the compressed gas flow, the Membrane Oxygenator was maintained at 40 °C by using a T9 Smart Cup/Bottle Warmer (Diyeeni, Item no. Diyeenigz2optngfa, ASIN-B0F1SLZNZM). As the medium left the Membrane Oxygenator and traversed the tubing it cooled to 37 °C matching the bore temperature (Figure S2).

### RTPC NMR Spectroscopy

NMR experiments were performed as described.^17^ Briefly, two types of experiments were performed in triplicate using biological replicates to assess the metabolic state of the cells. To identify and assign metabolites derived from the isotopically labeled glucose a continuous 100–200 μL/min flow of [U–^13^C]-glucose in KH medium was used. A proton-decoupled ^31^P spectrum was collected, followed by a 2D ^1^H–^13^C heteronuclear single quantum coherence, HSQC, experiment, then another ^31^P spectrum. To measure metabolic kinetic parameters, a 15 mL ^13^C-glucose pulse in KH medium was introduced through the in-line injection loop at 100–200 μL/min. Prior to injection a proton-decoupled ^31^P spectrum was collected, followed by the pulse during which 500 1D ^1^H–^13^C HSQC spectra, also called ^13^C isotope-edited ^1^H spectra, were acquired, then a final ^31^P spectrum. For each experiment, a ^13^C isotope-edited ^1^H spectrum was collected prior to the injection of the ^13^C-glucose to establish a baseline reading of the metabolites present in the sample due to natural ^13^C abundance.

All NMR spectra were recorded at 310 K using a 600 MHz Bruker Avance III NMR spectrometer equipped with a QCI-P cryoprobe. ^31^P spectra were recorded with 3000 scans and a 1 s recycle delay, centered at −10 ppm, corresponding to 2429.37 Hz, and a spectral width of 39 ppm. Peak intensities at 5.5, 10.6, and 19.1 ppm, which correspond to the γ-, α-, and β-phosphates of ATP, were used to assess cellular health and viability.^26,28,29^ If the ATP peaks were at noise level, the cells were determined to lack viability and were not used. 2D 1H–^13^C HSQC spectra were collected using a standard Bruker pulse sequence hsqcetgpsp.3 to detect metabolites incorporating ^13^C from [U–^13^C]-glucose. The phase sensitive pulse sequence uses echo-antiecho method and adiabatic pulses for inversion and refocusing and compensation of Bloch-Siegert effects.^30^ Spectra were collected with 32 scans, centered at 4.7 and 44 ppm and digitized by 2048 and 512 points in the ^1^H and ^13^C dimensions, respectively. The spectral widths in the ^1^H and ^13^C dimensions were 16 and 80 ppm, respectively. Metabolite kinetic profiles were generated by acquiring 1D ^13^C-edited ^1^H spectra using 32 scans. The spectral widths in the ^1^H and ^13^C dimensions were 16 and 80 ppm, respectively, and were digitized by 2048 points and 1 point in the ^1^H and ^13^C dimensions, respectively. The time of acquisition for each transient was 47 s. After introducing the pulse, 500 spectra were collected consecutively over a 6.5 h period allowing the disposition of the [U–^13^C]-glucose to be monitored as it progressed through different metabolic pathways. All spectra were processed by using Topspin 4.2.0 (Bruker).

### NMR Data Analysis

NMR spectra were processed to remove baseline noise and outliers followed by 10-point adjacent averaging to smooth the pulses. Each pulse was scaled and normalized to enable direct comparison between replicates. The analysis of metabolite kinetic profiles was as previously described^17^ with the following exceptions. The KH buffer was supplemented with palmitic acid from an ethanolic stock. The ethanol peak at 1.23 ppm was used as an internal reference to normalize the 1D spectra (Figure S3). Peak intensities were calculated as *I* = (*I*_*n*_/*I*_EtOH_*n*_), where n is the experimental scan index. Two dimensional ^1^H–^13^C HSQC contours were normalized using the ^13^C-glucose anomeric proton cross peaks at 5.2 ppm in the ^1^H and 92.3 ppm in the ^13^C dimensions. The one-phase association model from GraphPad Prism was used to fit the leading and trailing edges of the kinetic profiles to provide estimates for the production time, *t*_p_, and clearance time, *t*_c_, for glucose, lactate, alanine and glutamate.

$$Y=Y_{o}+\left(Plateau-Y_{o}\right)\left(1-\exp\left(-Kx\right)\right)$$

### Lactate Control Experiment

Undifferentiated and differentiated SH-SY5Y cells were grown as described above on plates. Cells were treated with KH medium containing ^12^C-glucose for 30 min, followed by the introduction of [U–^13^C]-glucose. Five hundred μL extracellular samples were collected at 2 min intervals for 30 min and ^13^C-edited 1D ^1^H spectra were acquired to monitor the rise in excreted lactate. Lactate peak volumes were fit to an exponential growth curve to resolve the time of lactate accumulation.

## Results

### Differentiation of SH-SY5Y Cells

The procedure used to differentiate SH-SY5Y cells entailed sequential incubation of the cells in the presence of retinoic acid (RA) and tetradecanoyl phorbol acetate (TPA) and was slightly modified from previously reported protocols.^5,24^ The changes were dictated by the need to minimize perturbation of the cells to maximize metabolic processes and to minimize contamination that may arise early in the lengthy incubation process. The primary modifications included frequent, 12 h changes in growth medium and a shortened incubation period. Cells were differentiated on plates and in alginate beads for imaging and dopamine analysis and in alginate beads for RTPC-NMR. Plate-differentiated SH-SY5Y cells are shown in Figure 1A. The presence of thick, branching neuronal processes confirmed that the modified differentiation protocol was successful.

**Figure 1:**
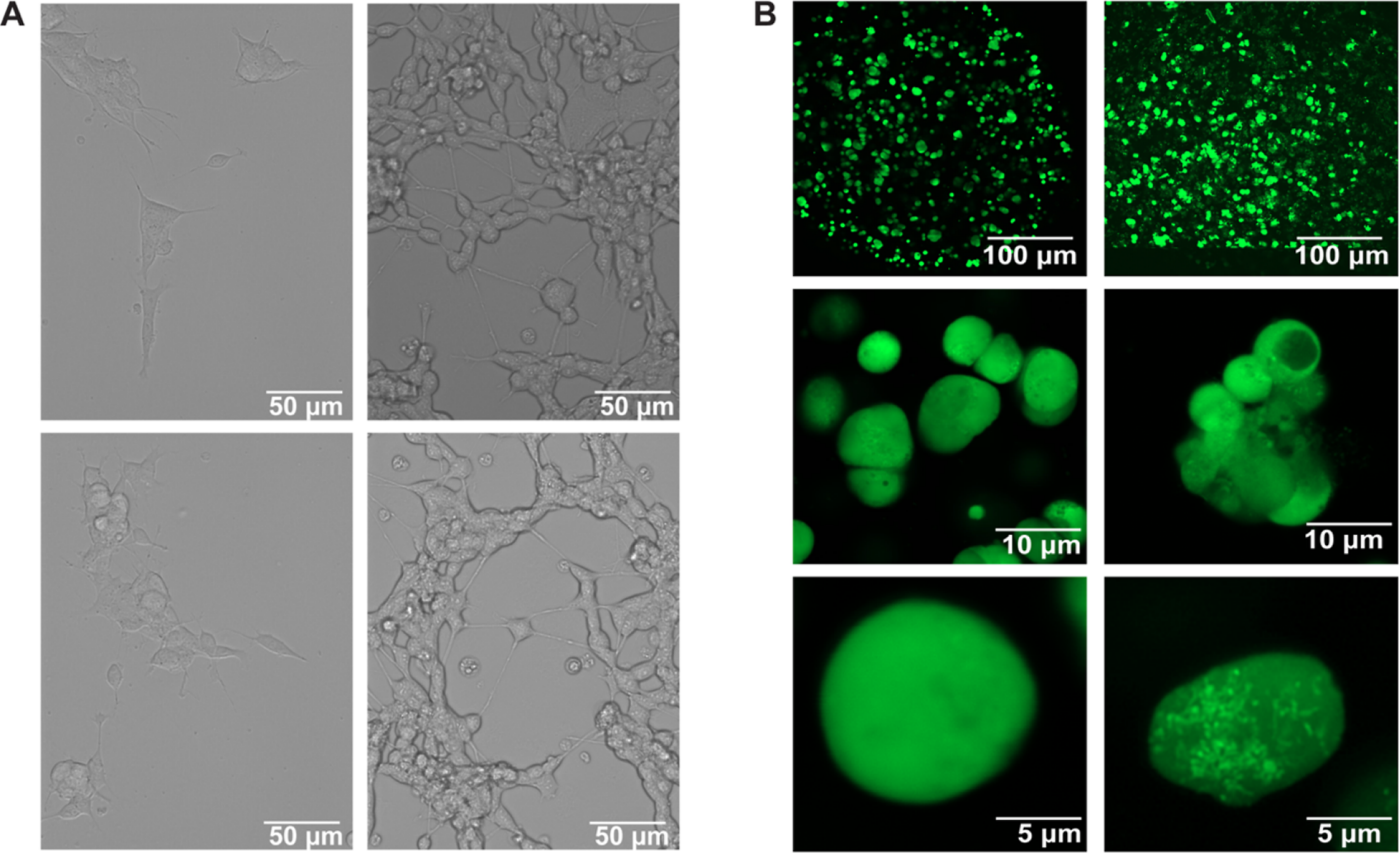
Morphology of differentiated SH-SY5Y cells. (A) Left panels show two images of undifferentiated cells 96 h after plating. Right panels show two images of cells following the 96 h plate-differentiation protocol. (B). Fluorescence microscopy of calcein-AM-stained undifferentiated (left) and differentiated (right) SH-SY5Y cells in beads.

To effectively study changes in metabolism accompanying dopaminergic differentiation of neuronal cells by RTPC-NMR spectroscopy, cells need to be packaged into and differentiated in alginate beads prior to loading into the NMR bioreactor.^17^ The procedure for casting alginate beads was modified to maintain sterility by filtering the air used in the atomizer (Figure S1). Type I bovine collagen was added to the alginate to provide a matrix for cell adhesion during differentiation.^31^ Once encapsulated, the cells were differentiated using the same protocol as for plate differentiation with one change: In addition to penicillin and streptomycin, kanamycin was included in the growth medium.

Packaged, differentiated SH-SY5Y cells are not expected to mirror the same morphology as cells differentiated on tissue culture plates. The turgor pressure required to produce classic neuronal morphology is exceeded by the density of the alginate-collagen matrix, which will limit the expansion of neuronal processes. Beads were stained with calcein AM to assess cell viability and the extent of differentiation. Nonfluorescent calcein AM is hydrolyzed in the cytoplasm of living cells by intracellular esterases into green fluorescent calcein. Fluorescent imaging showed the presence of viable cells encapsulated within alginate beads (Figure 1B). Close up images of the beads showed distinct differences between differentiated and undifferentiated cells (Figure 1B, middle and bottom). Stippled staining indicated the presence of neuronal processes in the beads containing differentiated cells. These observations support the fact that the cells successfully differentiated in the beads and remained viable despite the apparent morphological differences between cells differentiated on plates and in alginate beads.

### Dopamine Expression in Differentiated Cells

Increased dopamine production and dopamine receptor expression in differentiated SH-SY5Y cells is well described.^5,24,32,33^ Competitive ELISAs were used to assess whether or not the modified differentiation protocol successfully induced a dopaminergic phenotype, which is critical for metabolic and kinetic analyses by using in-cell NMR. Because this is a competitive assay the absorbance values decreased with increasing amounts of dopamine (Figure S4). Dopamine levels in bead lysates were lower than lysates from plated cells, which may be related to cell stress and reduced access to nutrients in the alginate-collagen matrix (Figure 2). However, under both plated and packaged conditions, differentiated cell samples showed a statistically significant (*p* < 0.0001) increase in dopamine concentration; 7-fold and 8-fold respectively, showing that the abbreviated protocol was effective, again despite the morphological differences observed between cells differentiated on plates and in beads.

**Figure 2:**
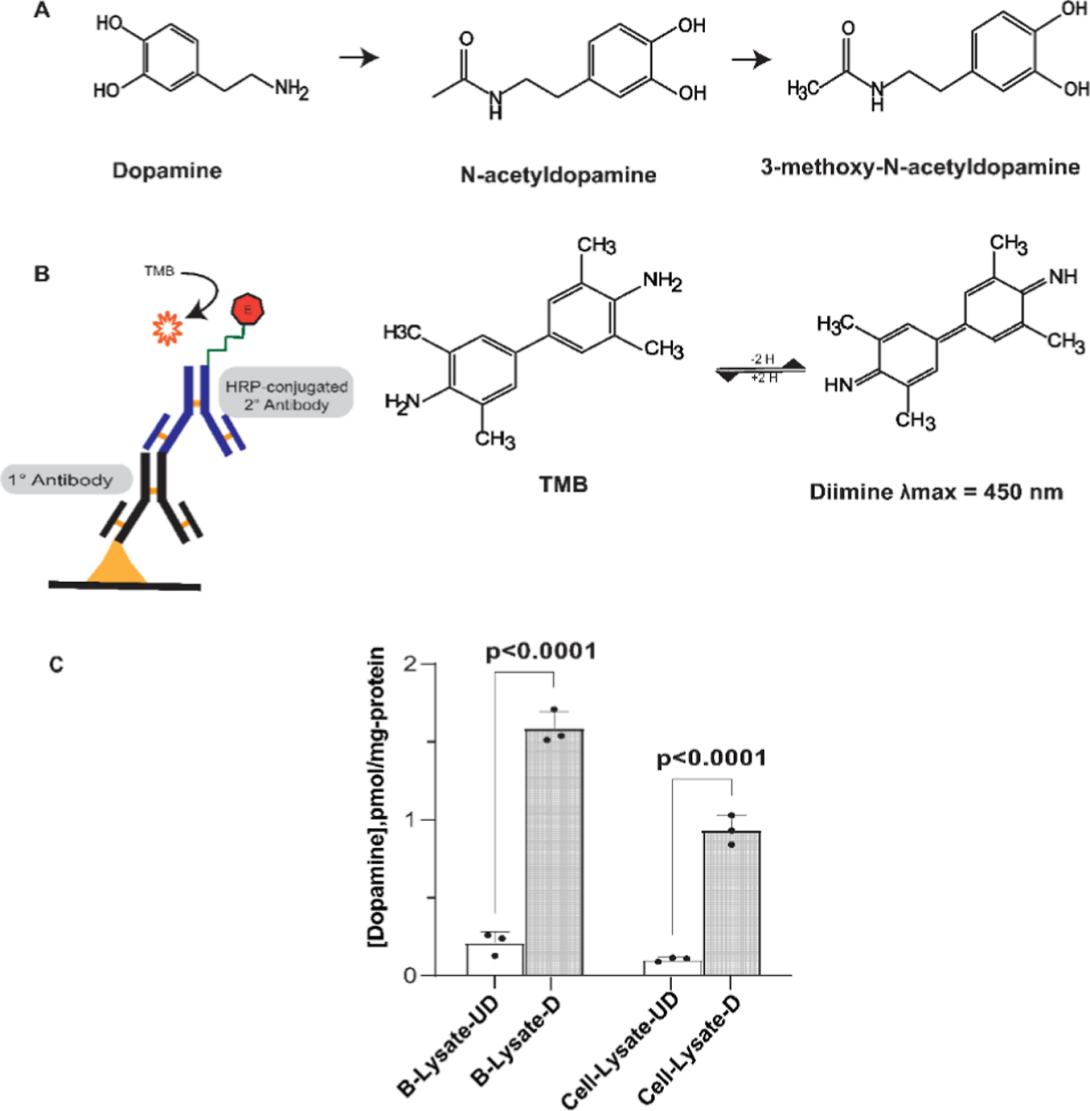
Neuronal differentiation enhances dopamine production. (A). Intracellular dopamine was captured on boronate affinity plates, acylated, and enzymatically methylated. (B). Competitive ELISA detects dopamine using an antidopamine antibody and an HRP-conjugated secondary antibody using a chromogenic substrate 3,3′,5,5′-tetramethylbenzidine (TMB). (C). Dopamine concentrations (pmol/mg protein) from differentiated (D) and undifferentiated (UD) bead (B) and cell lysates. Error bars indicate standard deviation.

### Undifferentiated and Differentiated Neurons Have Different Metabolic Profiles

One of the primary challenges in adapting in-cell NMR for neurons is the rapid decline in oxygen levels under static conditions, which can lead to cellular stress and deterioration of metabolic signals.^34,35^ To mitigate this, an oxygenation module was incorporated into the bioreactor (Figure S2). The module minimized turbulence and bubble formation, both of which can be detrimental to neuronal health and NMR data acquisition. Heating the oxygenator to 37 °C ensured that the perfused medium remained at physiological temperatures, further supporting cell viability during lengthy NMR experiments.

To assess differences in the metabolic profiles between undifferentiated and differentiated cells, alginate beads were loaded into the bioreactor and equilibrated prior to introducing a continuous flow of isotopically labeled ^13^C–glucose (Figure 3A). Proton-decoupled one-dimensional ^31^P spectra were recorded for the first and last 45 min of each experiment to assess cell viability (Figure S5A,B). ATP levels remained steady over the course of each 7 h experiment but were ∼50% lower for differentiated cells (Figure S5C). The drop in energy charge indicates a loss of cell viability in alginate beads. This was not unexpected given the long residence time of the cells within the beads, i.e. five days from encapsulation until the end of the NMR experiment, and the reduced diffusion of nutrients into and waste out of the interior-most bead volume. Despite the ∼50% reduction in ATP levels, the ATP signal-to-noise ratio remained above baseline and revealed clear metabolic differences between undifferentiated and differentiated cells (Figure S5), showing that the system remained informative despite the energetic stress.

**Figure 3:**
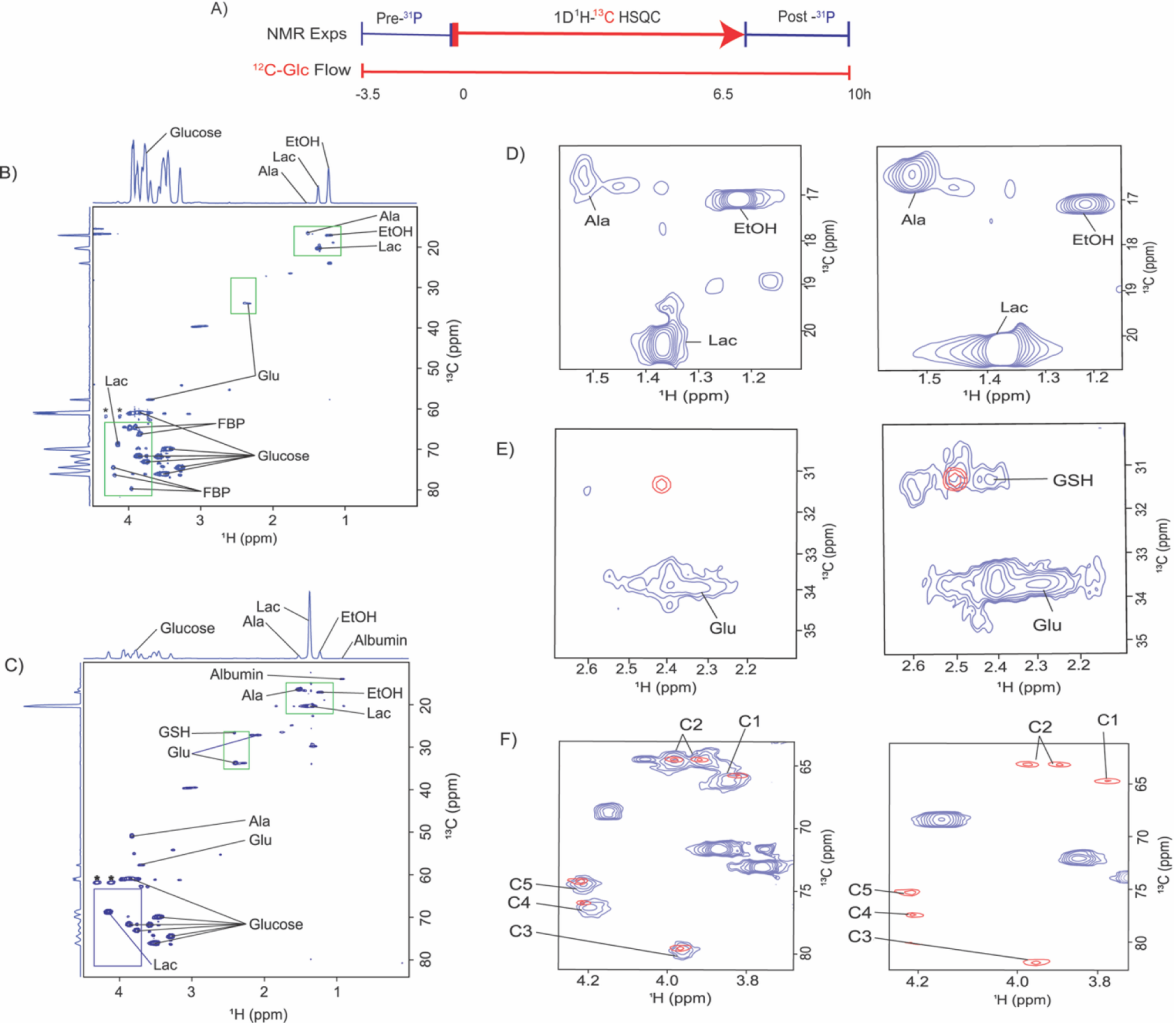
Undifferentiated and differentiated SH-SY5Y cells have distinct metabolic profiles. (A). Experimental time course for continuous flow of ^13^C-glucose (Glc). (B). Representative 2D ^1^H–^13^C HSQC spectrum of undifferentiated SH-SY5Y cells showing strong signals for glycolytic metabolites, lactate (Lac), alanine (Ala), fructose-1,6-bisphosphate (FBP) and glucose. (C). 2D ^1^H–^13^C HSQC spectrum of differentiated SH-SY5Y cells showing glutathione (GSH) and increased signals for glutamate (Glu) and Ala. The projection of the 2D ^1^H–^13^C HSQC into the proton dimension is shown on the top of each spectrum. Asterisks indicate unassigned peaks. (D). Close-up of the boxed areas in (B) (left) and (C) (right) showing Lac, Ala and EtOH cross peaks. (E). Close-up of the boxed areas in (B) and (C) showing Glu and GSH cross peaks. (F). Close-up of the boxed areas in (B) and (C) showing FBP cross peaks.

Two dimensional ^13^C-edited heteronuclear single quantum coherence, HSQC, NMR spectra revealed distinct differences between undifferentiated and differentiated cells (Figure 3B,C). The ^13^C–^13^C couplings observed under continuous flow confirmed that the metabolic products were catabolized from uniformly ^13^C-labeled glucose, [U–^13^C]-glucose. Ala and Glu peaks were more prominent in differentiated cells, suggesting an increased reliance on the TCA cycle. The cross peak splitting observed in the carbon dimension, especially in differentiated cells, showed that lactate, alanine and glutamate were products of glycolysis and the TCA cycle (Figure 3D,E). Glutathione, GSH, an important antioxidant, was observed in differentiated cells but undetectable in undifferentiated cells (Figure 3E). The GSH ^13^C–^13^C coupling is shown in Figure S6. The most notable observation was the disappearance of observable fructose 1,6-biphosphate, FBP, in differentiated cells (Figures 3F, and S7).

The simultaneous absence of FBP and the emergence of GSH in differentiated cells signify profound metabolic reprogramming. Undifferentiated cells engage in aerobic glycolysis rapidly converting pyruvate into lactate via lactate dehydrogenase, LDH, a hallmark of the Warburg effect,^36^ in which cells rely on glycolysis rather than oxidative phosphorylation for energy production even when oxygen is available, and indicative of reduced mitochondrial respiration, while the slower anabolic lactate production kinetics observed in differentiated cells showed a greater reliance on mitochondrial oxidative metabolism, i.e. the TCA cycle, for energy production. Collectively, these findings confirm that differentiation drives a key metabolic transition from a glycolytic, tumor-like phenotype to an oxidative, neuron-like phenotype.

### Cell Differentiation Alters Metabolite Kinetics

A series of rapidly acquired, 47 s interval,^17^ 1D NMR spectra were used to measure metabolite kinetics. Because proton signals from metabolites overlap, 1D ^13^C-edited proton NMR experiments were used to monitor carbon that was metabolized from the isotopically labeled glucose. RTPC-NMR was used to monitor the monitor the time-dependent metabolic response to the [U–^13^C]-glucose pulse, as it was incorporated into synthetic pathways to produce Lac, Ala and Glu. Each experiment consisted of 500 1D ^13^C-edited proton NMR spectra that took ∼6.5 h to collect, sandwiched between two 1.5 h ^31^P experiments to assess cell viability (Figure 4A).^17,37^ Metabolic kinetic profiles for biological replicates of undifferentiated and differentiated cells are shown in Figure 4. The leading and trailing edges were fit to a one-phase association or decay curve, respectively, to yield a production time, t_P_, that quantifies the rate of incorporation of the ^13^C label into each synthesis pathway following initiation of the pulse, and a clearance time, t_C_, that quantifies the rate at which the metabolic label is removed following initiation of the chase (Figures S8 and S9; Table 1).^17,38,39^

**Figure 4:**
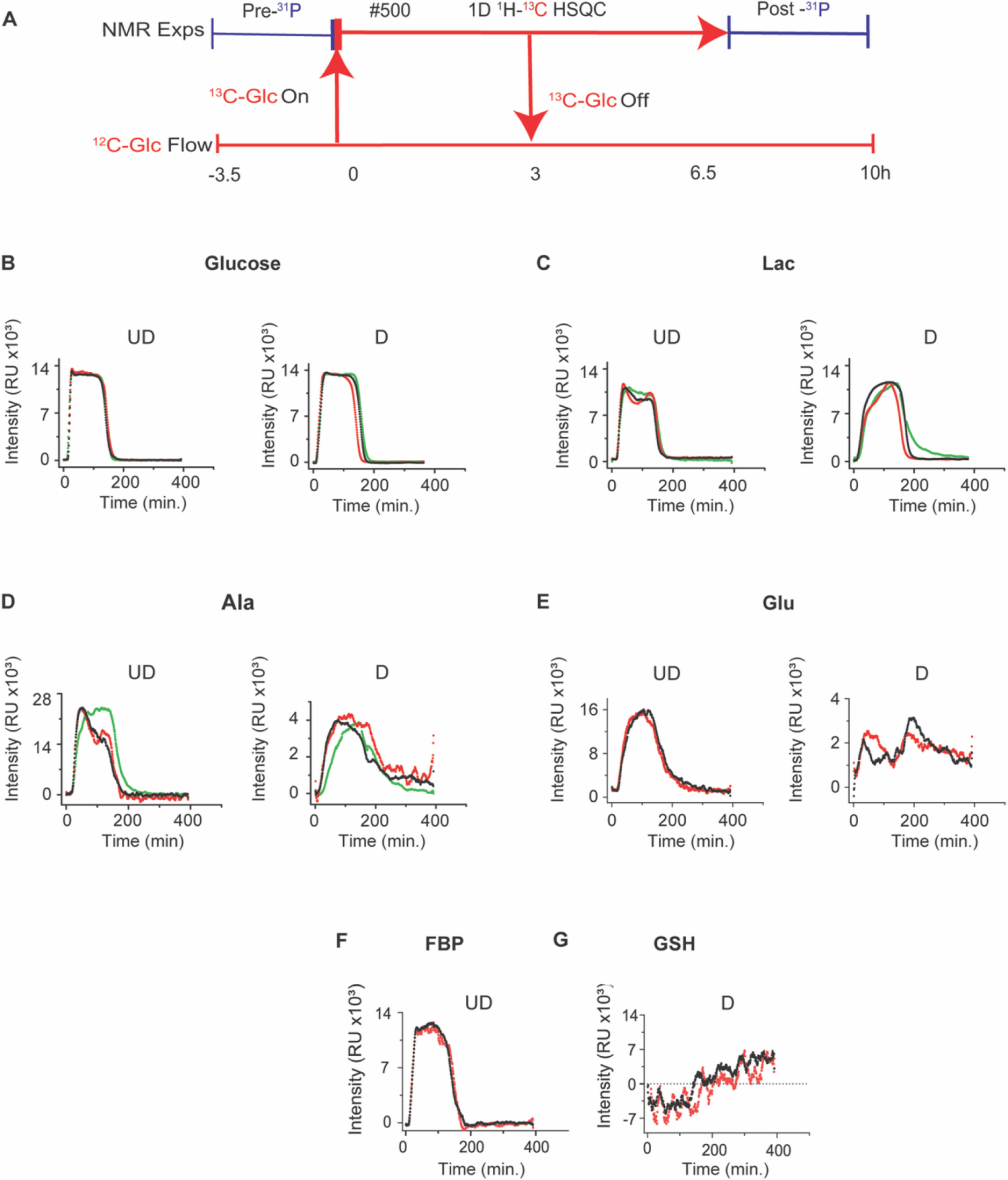
RTPC-NMR profiles of metabolites in undifferentiated and differentiated SH-SY5Y cells. Red, black, and green traces represent individual biological replicates from experiments performed in triplicate for glucose, lactate (Lac) and alanine (Ala), glutamate (Glu), fructose 1,6-biphosphate (FBP) and glutathione (GSH). For Glu, FBP and GSH only two replicates were analyzable. (A). Experimental time course for RTPC-NMR spectroscopy using ^13^C-glucose (Glc). (B). [U–^13^C]-glucose pulse chased by ^12^C-glucose showing the perfusion of the label through the bead matrix containing undifferentiated and differentiated cells. (C–E). Kinetic pulse profiles for Lac; Ala and Glu in undifferentiated and differentiated cells. (F). Kinetic pulse profile for FBP in undifferentiated cells. (G). Kinetic pulse profile for GSH in differentiated cells.

**Table 1:** Average Production, *t*_p_, and Clearance, *t*_c_, Times of Metabolites

	undifferentiated		differentiated	
	*t*_p_ (min)	*t*_c_ (min)	*t*_p_ (min)	*t*_c_ (min)
Lactate	8.76 ± 2.0	14.0 ± 1.0	26.7 ± 1.1	20.0 ± 6.0
Alanine	12.0 ± 2.0	26.0 ± 2.0	37.0 ± 7.0	46.0 ± 4.0
Glutamate	25.1 ± 1.7	48.0 ± 1.7	14.0 ± 0.1	82.0 ± 0.4
FBP	11.1 ± 0.2	18.1 ± 0.1		

The leading and trailing edges of the glucose curves are comparable for both cell types confirming rapid delivery and clearance of ^13^C-glucose in the NMR-visible region of the bead matrix (Figure 4B). The average production times for glucose, 4.93 min in undifferentiated cells and 9.90 min in differentiated cells (Figure S9) represent a limit to the fastest kinetics that can be measured. However, the fastest average production time measured in these experiments, *t*_p_ = 8.76 min for lactate in undifferentiated cells (Table 1), was almost twice as slow. Thus, the limiting condition is not reached and the measured rates can be considered to provide a reliable basis for comparing relative metabolic responses under the conditions used in this study.

Distinct differences were observed between undifferentiated and differentiated cells for the downstream metabolite profiles. Two additional metrics were calculated to evaluate the metabolic kinetic profiles: the characteristic time, *t*_ch_ = 2/(1/*t*_p_ + 1/*t*_c_), defined as the harmonic mean between t_P_ and t_C_;^39^ and the binding parameter, *K* = (1/*t*_p_ – 1/*t*_c_)/2, which reflects the rates of incorporation of the pulse label into and release from the bound fraction of metabolite. The bound fraction includes the formation of any short- or long–term complex such as glutamate in storage vesicles or glucose incorporation into glycogen. *K* will be a small positive value unless the rate of clearance exceeds the rate of production. Larger *K* values indicate that *t*_c_ is much greater than *t*_p_, and a greater quantity of metabolite is sequestered in complexes (Table 2).^17^

**Table 2:** Characteristic Times, *t*_ch_, and Binding Constants, *K*, of Metabolites

	undifferentiated		differentiated	
	*t*_ch_ (min)	*K* (min^–1^)	*t*_ch_ (min)	*K* (min^–1^)
Lactate	11.0 ± 1.0	0.025 ± 0.01	22.0 ± 3.0	–0.016 ± 0.01
Alanine	15.0 ± 1.0	0.020 ± 0.01	40.0 ± 5.7	0.003 ± 0.00
Glutamate	33.0 ± 2.0	0.010 ± 0.00	24.0 ± 0.1	0.030 ± 0.00
FBP	14.0 ± 0.2	0.020 ± 0.00		

Undifferentiated cells exhibited a rapid rise in lactate to peak levels, *t*_p_ = 9.0 min, that remained elevated for a short duration before clearing rapidly, *t*_c_ = 14.0 min. In contrast, differentiated cells showed a much slower rise, *t*_p_ = 26.7 min, that cleared as quickly as in undifferentiated cells, *t*_c_ = 20.0 min, after reaching an intensity comparable to what was observed in undifferentiated cells (Table 1). The differences in kinetics were echoed by the short characteristic times resolved for undifferentiated cells, 11.0 min, vs 22.0 min, for differentiated cells (Table 2, Figure 5). In addition, the binding constant shifted from a positive value in undifferentiated cells, *K* = 0.025 min^–1^, to a negative value in differentiated cells, indicating a very rapid clearance rate relative to production. A negative value for *K* supports the idea that Lac is rapidly exported from the system and is less likely to be stored in differentiated cells. A negative characteristic binding constant was previously observed for glutamate metabolism in HEK293T cells.^17^ These findings align with a shift from glycolytic metabolism to oxidative phosphorylation as cells adopt a neuron-like phenotype and reduce internal lactate usage.^3,4,40^

**Figure 5:**
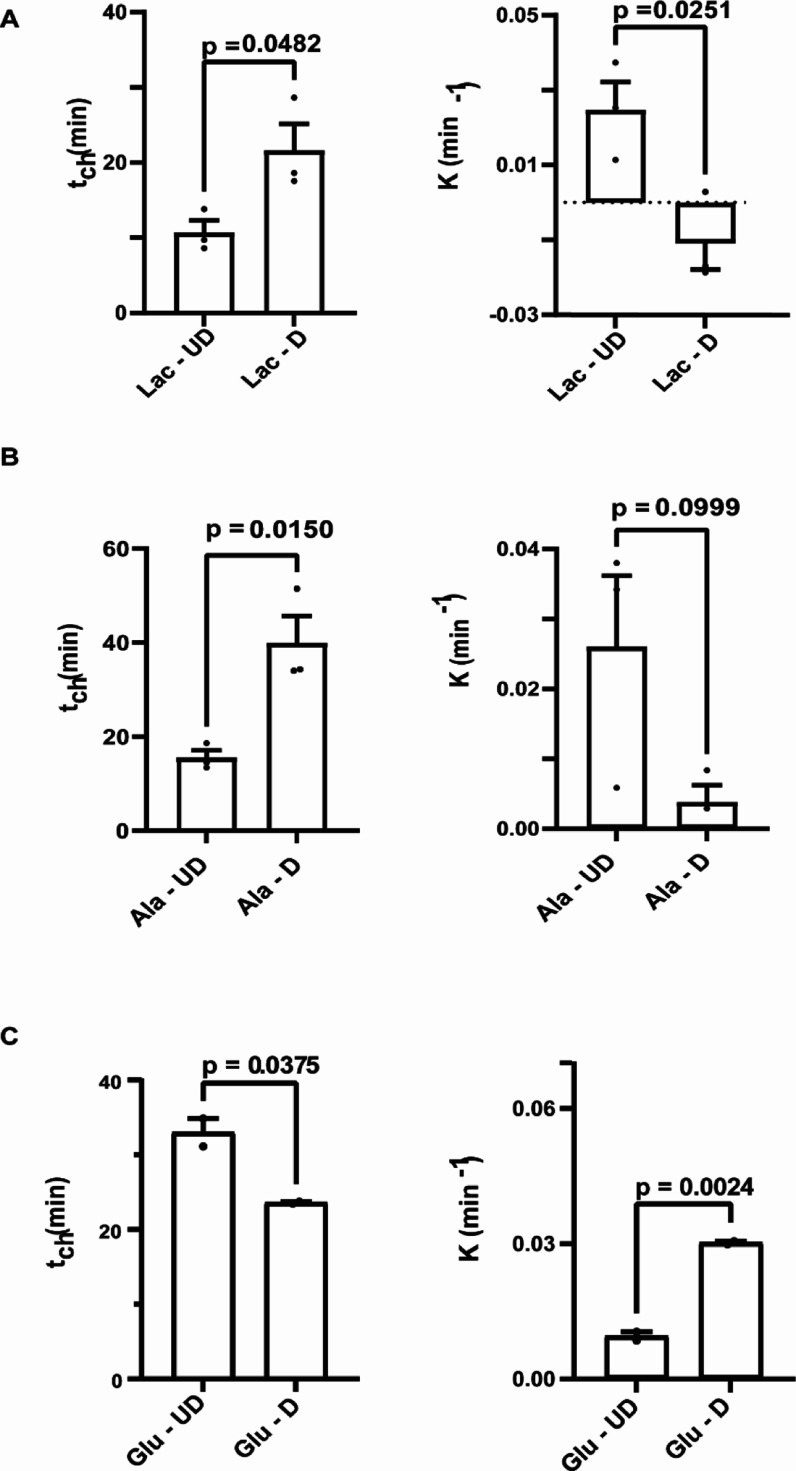
Changes in characteristic times, *t*_ch_, and binding parameters, *K*. (A) Lactate; (B) Alanine; (C) Glutamate in undifferentiated and differentiated SH-SY5Y cells. Error bars indicate standard deviation.

Alanine metabolism also differed between the two cell types. In undifferentiated cells, alanine levels increased rapidly, *t*_p_ = 12.0 min, comparable to that of lactate production, reaching a sharp peak that decreased slowly before taking about twice as long to clear than lactate, *t*_c_ = 26.0 min. In contrast, differentiated cells exhibited a slower increase in alanine intensity, *t*_p_ = 37.0 min, comparable to that of lactate, that peaked at ∼15% of the intensity level seen in undifferentiated cells and a much longer clearance time, *t*_c_ = 46.0 min, that did not return to baseline level over the course of the experiment (Table 1). There was a highly significant difference in the characteristic times resolved for alanine between undifferentiated and differentiated cells, *t*_ch_ = 15.0 vs 40.0 min, and a significant difference in the binding constant, 0.02 min^–1^ for undifferentiated cells, which was comparable to lactate, and 0.003 min^–1^, for differentiated cells (Table 2, Figure 5). These differences show that differentiated cells utilize alanine more slowly and imply a slow exchange of free alanine from a bound population that is maintained to meet sustained energy demands, consistent with neuron-like function. The reduced intensity of the alanine signal is also consistent with a substantial fraction of the population being transiently bound or incorporated into larger biomolecules. The results suggest that in differentiated cells alanine may act as a secondary metabolite or overflow byproduct, rather than playing a central role in active metabolic pathways.^41^

Notable differences were evident in the glutamate kinetics between the two cell types. In undifferentiated cells, glutamate levels took longer to reach maximum intensity and to clear than lactate and alanine, *t*_p_ = 25.1 and *t*_c_ = 48.0, reaching maximum intensity about the time the labeled glucose pulse was chased from the system (Table 1). The slower production times are characteristic of glutamate because of its dependence on the products of the TCA cycle and the slow import and export of metabolites to and from mitochondria. Clearance was complete, consistent with the rapid turnover typical of proliferating cells. In differentiated cells glutamate levels rose twice as fast as lactate and alanine, *t*_p_ = 14.0, to ∼15% of the maximum level observed in undifferentiated cells, then decreased before rising and falling again. Clearance was very slow, *t*_c_ = 82.0, remaining steady and failing to return to baseline intensity levels over the time course of the experiment. There was a significant difference in the characteristic times resolved for glutamate between undifferentiated and differentiated cells, *t*_ch_ = 33.0 vs 24.0 min, indicating faster production in differentiated cells and a highly significant difference in the binding constant, 0.010 min^–1^ vs 0.030 min^–1^, between undifferentiated versus differentiated cells indicating more rapid clearance of glutamate from undifferentiated cells and a more extensive bound population in differentiated cells than observed for lactate or alanine (Table 2, Figure 5). The rapid synthesis and extended retention of glutamate in differentiated cells is consistent with the role of glutamate as a neurotransmitter precursor in mature neuron cells where it may be compartmentalized for synaptic signaling, neurotransmitter cycling and mitochondrial energy metabolism, characteristic of mature neuronal metabolism.^42,43^

The production, *t*_p_ = 11.1, clearance, *t*_c_ = 18.1, and characteristic times, *t*_ch_ = 13.8, for FBP were fast, comparable to the turnover rates of glycolytic products, lactate and alanine, in undifferentiated cells (Figure 4, Table 1). The binding constant, *K*, was close to zero indicating very rapid clearance relative to production and consistent with the high glycolytic turnover associated with undifferentiated cells. In differentiated cells increasing GSH production was observed throughout the pulse and chase phases of the experiment (Figure 4). Continuous synthesis of GSH is likely a response to counter ROS production from the predominance of oxidative phosphorylation in these cells. In addition, the observation of a bound population of glutamate provides a continuous reactant for biosynthesis of GSH, which requires cysteine and glycine as well.

As an independent control to evaluate the effects of encapsulation and the bioreactor on cell metabolic stability, the rate of lactate excretion from unencapsulated undifferentiated and differentiated SH-SY5Y cells maintained on plates was measured (Figure S10). The cells performed comparably to encapsulated cells in two regards; (1) the onset of Lac production occurred on the same time scale, i.e. <30 min, as observed in the kinetic profile experiments, and (2) differentiated SH-SY5Y cells excreted more lactate, as has been previously observed.^44,45^ The high rate of lactate excretion is consistent with the rapid clearance time observed giving rise to a negative characteristic binding constant (Table 2). The experiment confirmed that the manipulations of encapsulation and insertion into the bioreactor did not compromise the vitality of the cells and supports the idea that the differences in metabolism observed arise from the differentiated cellular state.

## Discussion

Earlier studies, which were based on measurements of cell extracts, proposed that neuronal differentiation involves a fundamental shift in primary metabolism from glycolysis to oxidative phosphorylation.^3,40^ This hypothesis was supported by transcriptomic analyses, static measurements of metabolite concentrations and inferred shifts in metabolic gene expression.^3,40^ In addition, decreased expression of glycolytic genes such as lactate dehydrogenase A hexokinase 2, a switch in pyruvate kinase isoforms from PKM2 to PKM1,^3,40^ and relatively stable expression levels of TCA cycle genes collectively suggested an increased reliance on mitochondrial function.^3,40^

In-cell NMR spectroscopy provides a powerful lens to study real-time neuronal metabolism, allowing the study of living cells without disrupting their internal environment.^17^ Unlike traditional approaches such as RNA sequencing,^46^ gas chromatography–mass spectrometry^47^ or oxygen consumption assays that disrupt the cells, RTPC-NMR continuously tracks the incorporation of isotopically labeled ^13^C-glucose into key downstream metabolites on a subminute time scale while preserving cellular viability and provides kinetic data on the real-time metabolic transitions occurring within intact neurons. In this work, SH-SY5Y neuroblastoma cells were encapsulated and differentiated into dopaminergic neuronal cells within biocompatible alginate-collagen beads, the metabolic profiles of the cells were characterized and the kinetic parameters of several metabolites were evaluated. The results revealed that distinct metabolic reprogramming accompanies differentiation: Undifferentiated cells engaged in aerobic glycolysis while the slower anabolic activity for lactate observed in differentiated cells showed a greater reliance on mitochondrial oxidative metabolism, which is dependent on the products of glycolysis for energy production (Figure 6). The results revealed extended retention of glutamate, highlighting its increased functional relevance in neurotransmitter cycling and mitochondrial energy metabolism, an indicator of mature neuronal metabolism.^42^

**Figure 6:**
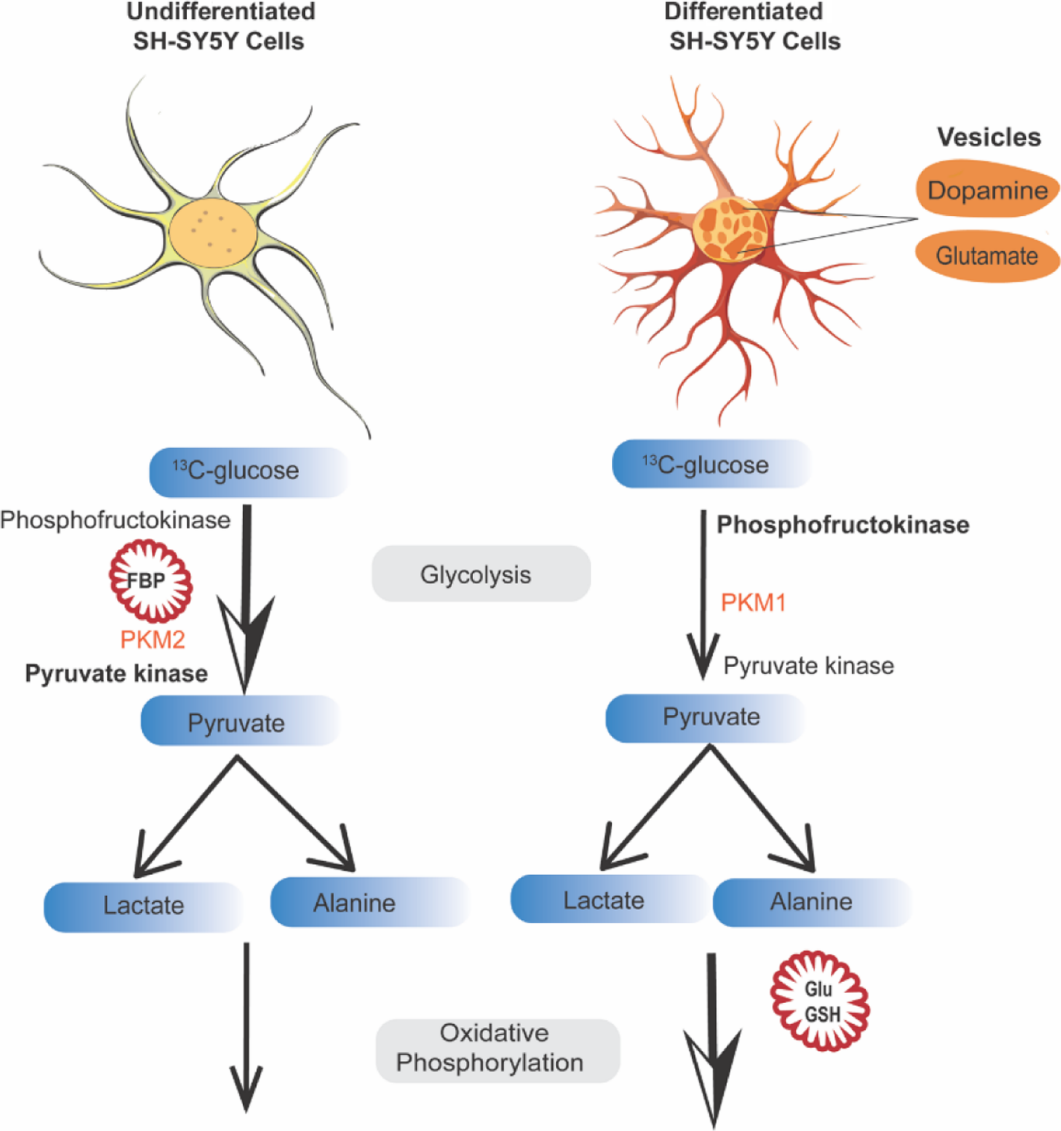
Metabolic profiles of undifferentiated and differentiated SH-SY5Y cells. In undifferentiated cells, glycolysis is the dominant energy production pathway. Expression of the less active pyruvate kinase M2 isoform, PKM2, may promote the accumulation of glycolytic intermediates such as FBP. The accumulation of FBP implies that pyruvate kinase is the rate-limiting step in undifferentiated cells. In differentiated cells, oxidative phosphorylation is the dominant pathway. Differentiated cells produce dopamine and accumulate glutamate and glutathione. Dopamine and glutamate are likely stored in vesicles. Expression of highly active PKM1 restores glycolysis to steady-state levels with phosphofructokinase as the rate-limiting step.

Increased glutamate synthesis suggests that differentiated SH-SY5Y cells generate the structural machinery required for neurotransmitter storage. This is consistent with earlier studies, showing the production of synaptic vesicles in RA-induced differentiation of SH-SY5Y cells.^48^ Further work showed that expression of vesicular glutamate transporters, which regulate loading of vesicles in glutamatergic neurons, is endogenously regulated.^24,49^ The results demonstrate that SH-SY5Y cells can express the essential transporters required to load and store vesicular glutamate.

Glutathione levels increased significantly in differentiated cells. GSH increases redox capacity enabling better management of ROS, which are produced in larger amounts in oxidative phosphorylation than in glycolysis. Higher GSH concentrations have been shown to be necessary for differentiated neuronal cell survival.^50^ Conversely, a decrease in intracellular GSH in neuronal cells and GSH depletion in cerebral granular neuron mitochondria have been linked to cell death.^51^ It has been proposed that glutathione dysfunction may be associated with neurodegenerative disorders such as Alzheimer’s disease,^52^ Parkinson’s disease,^53^ Huntington’s disease^54^ and amyotrophic lateral sclerosis.^55,56^ Thus, the presence of glutathione in differentiated SH-SY5Y cells is consistent with these observations. This protective adaptation, essential for neuronal survival, would have been missed in traditional metabolic analyses, underscoring the importance of RTPC-NMR’s real-time monitoring capabilities.

Notably, one of the most surprising observations was the appearance of FBP in undifferentiated cells and the complete disappearance in differentiated cells. This is consistent with previous observations showing that overexpression of PFK1 in human glioblastoma cells results in increased aerobic glycolysis,^57^ and in neural stem cells, which are phenotypically similar to SH-SY5Y,^6^ suppresses neural differentiation.^58,59^ Accumulation of FBP was also shown to inhibit oxidative phosphorylation in isolated rat mitochondria.^60^ FBP is the product of the rate-limiting step in glycolysis catalyzed by phosphofructokinase-1, PFK1. Glycolysis produces less ATP than oxidative phosphorylation resulting in lower intracellular ATP levels than would be present in cells utilizing oxidative phosphorylation for ATP production. PFK activity is increased at low intracellular ATP/AMP ratios, which may account for the increase in FBP levels.

FBP also plays a critical role in glycolysis by acting as a feedforward activator of pyruvate kinase M2, PKM2,^61,62^ which catalyzes the conversion of phosphoenolpyruvate into pyruvate. FBP activates PKM2 by inducing dimerization of inactive PKM2 dimers^63–65^ into active tetramers resulting in an increase in the production of glycolytic products lactate and alanine. An alternatively spliced isoform of PK, PKM1, also active in glycolysis, forms constitutive tetramers with greater activity than PKM2. The less active PKM2 can promote accumulation of glycolytic intermediates, in this case FBP. PKM2 is dominant in cancer and regulates the Warburg effect^66^ promoting the production of ATP without generating mitochondrial ROS. The switch from PKM2 to PKM1 expression is associated with the shift from aerobic glycolysis to oxidative phosphorylation. The increase in the activity of both PFK and PK in combination with the accumulation of FBP implies that in undifferentiated cells the rate-limiting step in glycolysis switches from PFK to PK. This differential regulation of the early and late stages of glycolysis may be key to understanding the switch in ATP production from glycolysis to oxidative phosphorylation that accompanies differentiation.

In conclusion, RTPC-NMR is a powerful platform for monitoring metabolism and the response to cellular stress under physiological conditions. The ability to differentiate cells within a gel matrix is a first step toward creating 3D models of neuronal tissue to examine the effect of neuroactive compounds on the physiology of neurons and critical to understanding and optimizing neurological therapies, in particular, those responsible for Parkinson’s and Alzheimer’s disease. The development of tools to apply to disease models will open a new venue in testing compounds and provide the opportunity to monitor functional response to drugs on the metabolic level, for which there is no precedent.

## Supplementary Material



## Notes

Additional images of cell casting in alginate beads and open-loop bioreactor setup, ethanol peak intensities, ELISA calibration curve, energy status of cells, NMR assignments of glutathione, glutamate, and FBP, kinetic flux analysis of undifferentiated and differentiated SH-SY5Y, and supplementary references (PDF)
