# Supplementary material for: Real Time Pulse Chase (RTPC) In-Cell NMR Spectroscopy Reveals Critical Metabolites in Subminute Metabolism of Undifferentiated and Differentiated Human Neuronal Cells
Source: Anal Chem. 2026 Jun 18;98(25):18373–85. doi: 10.1021/acs.analchem.5c07848 (PMC13325446; doi:10.1021/acs.analchem.5c07848)
Supplement: Supplementary file 1 [file ac5c07848_si_001.pdf]

Supporting Information

Real time pulse chase (RTPC) In-Cell NMR spectroscopy reveals critical metabolites in sub-minute metabolism of undifferentiated and differentiated human neuronal cells

Spoorthy Sandras[1], Nicholas Sciolino[1], Sergey Reverdatto[1], David S. Burz[1], Jayanti Pande[1], Ann Marie Schmidt[2], Ravichandran Ramasamy[2], Alexander Shekhtman[1*]

[1]Department of Chemistry, University at Albany, Albany, NY 12222, USA
[2]Diabetes Research Program, Department of Medicine, New York University Grossman School of Medicine, New York, NY, USA.
*Corresponding author, email: ashekhtman@albany.edu

Abstract

Neurons undergo extensive metabolic reprogramming during differentiation; this reprogramming leads to specific changes in the kinetics and concentrations of metabolites. We developed an in-cell NMR-based method that monitors this metabolic transformation with sub-minute time resolution. Undifferentiated SH-SY5Y human neuronal precursor cells were encapsulated into alginate gel beads and differentiated inside the gel. Real time pulse chase (RTPC) in-cell NMR was used to measure relative steady state concentrations of glycolysis and TCA cycle metabolites and the kinetics of metabolite production and clearance in differentiated and undifferentiated cells. Neuronal differentiation slowed glycolysis, increased TCA cycle activity and glutamate production. Fructose 1,6 bisphosphate and glutathione were identified as major biomarkers of undifferentiated and differentiated cells, respectively. The results demonstrate that RTPC-NMR analysis of neuronal cells is an effective method for studying changes in metabolite profiles induced by stress and drug-induced stimuli.

The supplementary information includes the following contents:

PAGE NUMBER	CONTENTS
S3	Figure S1. SH-SY5Y cell casting in alginate beads.
S4	Figure S2. Open-loop bioreactor for continuous delivery of oxygenated medium.
S5	Figure S3. Variation in ethanol peak intensities over the course of an RTPC-NMR experiment.
S6	Figure S4. ELISA calibration curve.
S7	Figure S5. Energy status of cells.
S8	Figure S6. NMR assignments for glutathione and glutamate from Figure 3E.
S9	Figure S7. NMR assignments for fructose 1,6-biphosphate from Figure 3F.
S10	Figure S8. Kinetic flux analysis of undifferentiated SH-SY5Y cells.
S11	Figure S9. Kinetic flux analysis of differentiated SH-SY5Y cells.
S12	Supplementary References

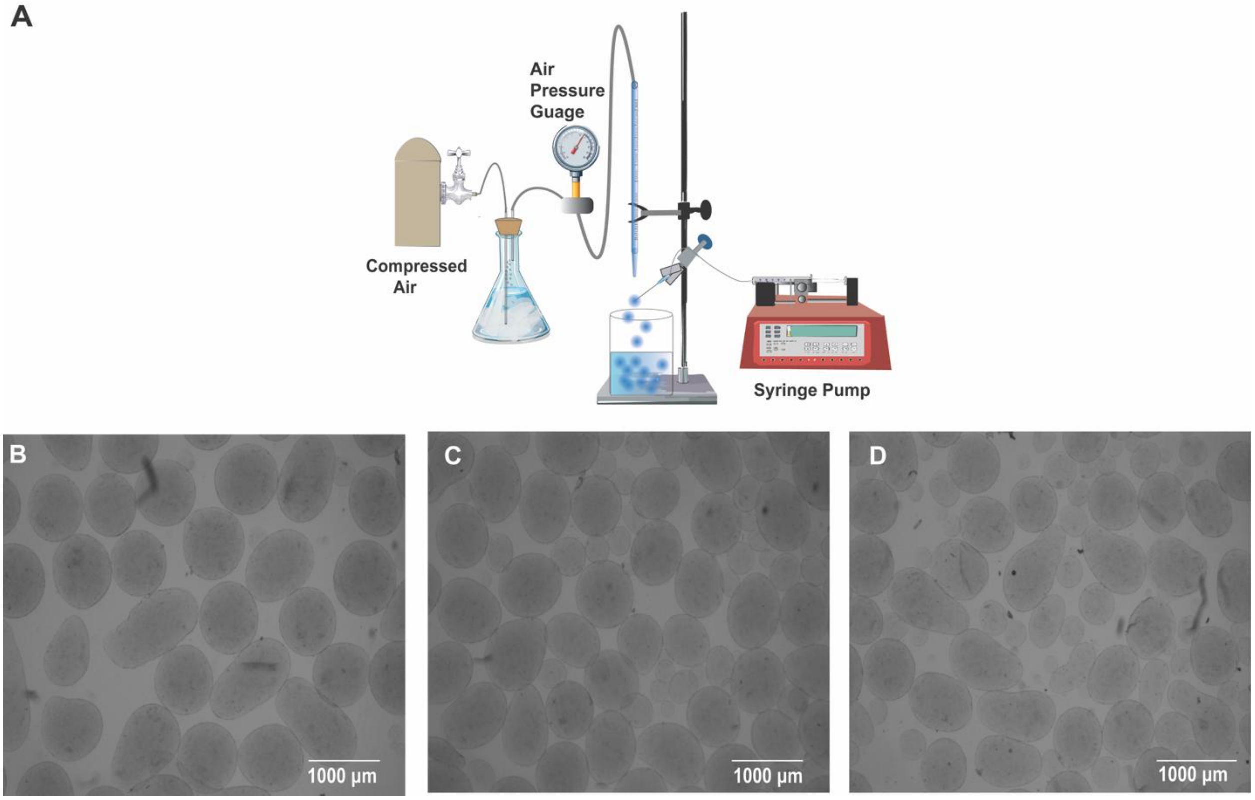

Figure S1. SH-SY5Y cell casting in alginate beads. A). Modified apparatus for casting SH-SY5Y cells in alginate beads for differentiation. Beads cast at B) 5.0 psi at 300 μL/min. C) 5.0 psi at 400 μL/min. D) 5.5 psi at 400 μL/min. The most uniform bead size and shape were obtained under conditions shown in Panel B.

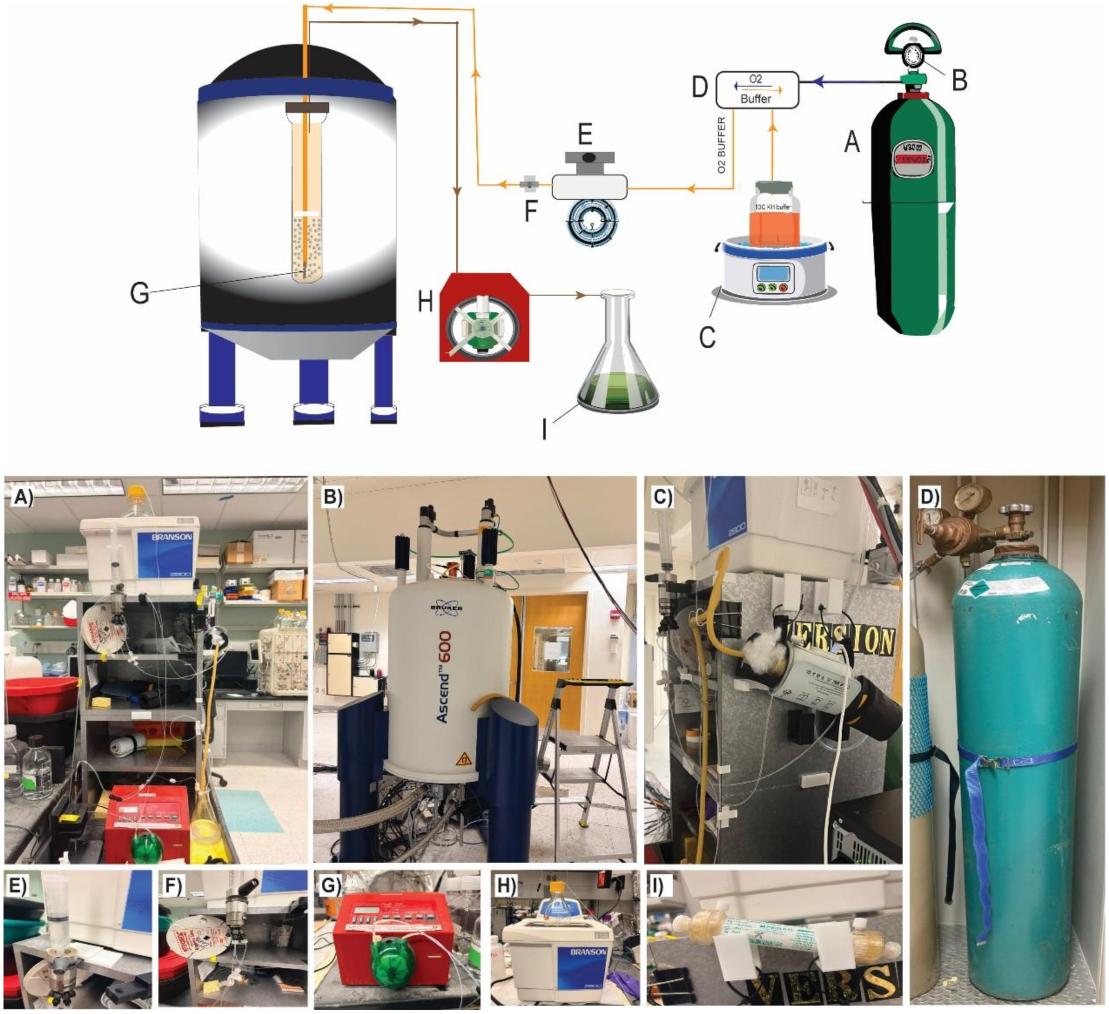

Figure S2. Open-loop bioreactor for continuous delivery of oxygenated medium. (Top) Schematic of experimental setup. A) Compressed gas mixture of **95% O_2/5% CO_2**. B) Pressure regulator/gauge. C). Medium reservoir in heated bath. D) D-150 membrane oxygenator wrapped with bottle warmer. E) Injector with 15 mL loop. F) Sampling/venting septum. G) NMR bioreactor tube with microporous diffuser. H). Programmable peristaltic pump. I). Waste collection**. (Bottom) Hardware used for in-cell NMR bioreactor experiments.** (A) Overview of the perfusion/bioreactor setup adjacent to the NMR spectrometer. (B) Bruker Ascend 600 NMR spectrometer used for data acquisition. (C) D-150 membrane oxygenator wrapped with bottle warmer. (D) Gas cylinder supplying the oxygenation mixture. (E, F) Close-up views of the flow and sample-handling connections. (G) Peristaltic pump used to control medium flow. **(H)** Branson ultrasonic cleaner used to pre-warm the medium. (I) Close-up of the membrane oxygenator. The photographs are provided to facilitate reproducibility of the experimental configuration.

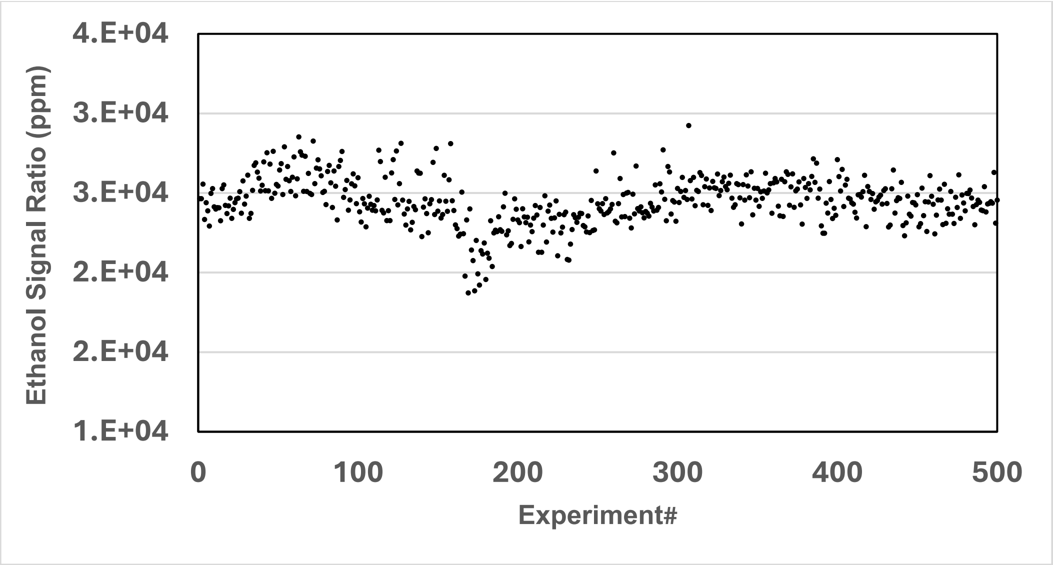

Figure S3. Variation in ethanol peak intensities over the course of an RTPC-NMR experiment. Ethanol peak intensities at 1.23 ppm were used as an internal reference to normalize 1D ^{13}C-edited ^{1}H spectra.

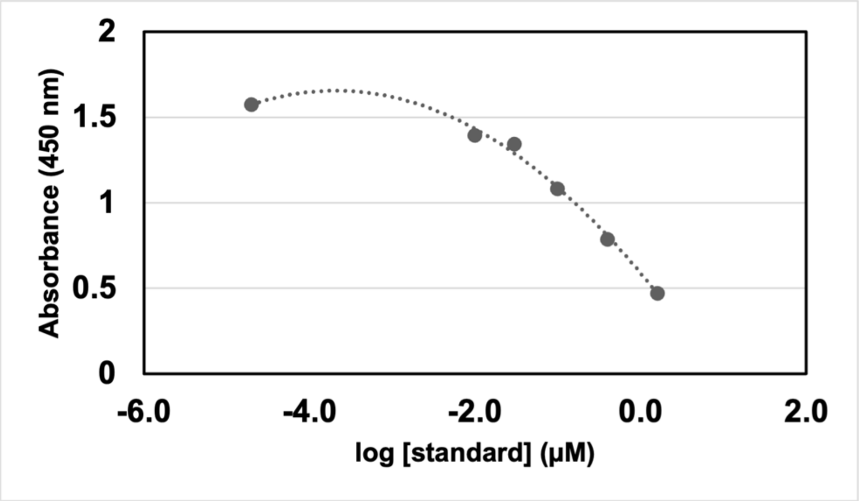

Figure S4. ELISA calibration curve. The relationship between absorbance and concentration was modeled using a non-linear regression fit to calculate the concentrations of unknown samples.

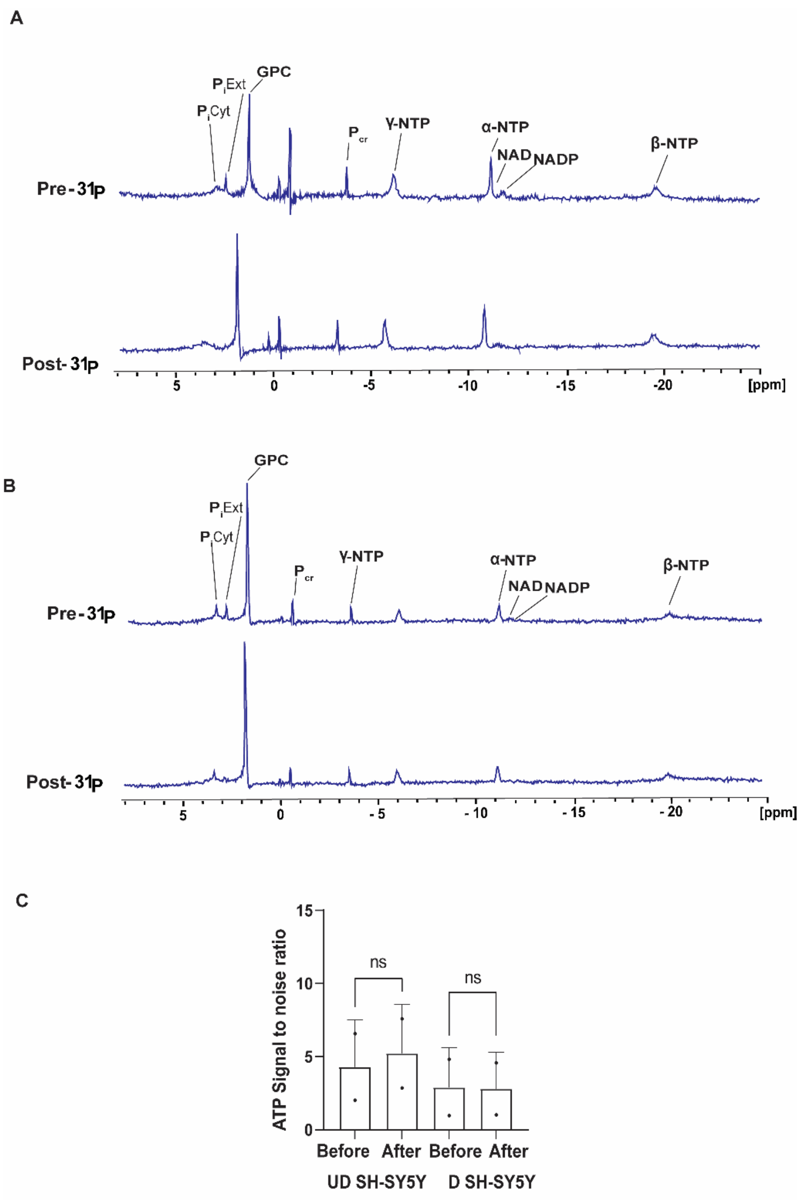

Figure S5. Energy status of cells. A). ^{31}P spectra of undifferentiated cells acquired prior to (top) and following (bottom) collection of 2D ^{1}H–^{13}C HSQC spectra. Peak assignments are indicated for P_{iEx}, extracellular inorganic phosphate; P_{iCyt}, cytosolic inorganic phosphate; PE, phosphatidylethanolamine; GPC, glycerophosphorylcholine; PCr, phosphocreatine; α-, β- and γ-NTPs; NAD, nicotinamide adenine dinucleotide and NADP, nicotinamide adenine dinucleotide phosphate. B). ^{31}P spectra of differentiated cells acquired prior to (top) and following (bottom) collection of 2D ^{1}H–^{13}C HSQC spectra. C). Signal to noise ratios of ATP levels for undifferentiated and differentiated cells. ATP signal-to-noise ratios were quantified by comparing the ATP peak intensity with baseline noise. The ATP signals in both undifferentiated and differentiated cells were greater than 3-fold above the baseline noise level, confirming reliable detection

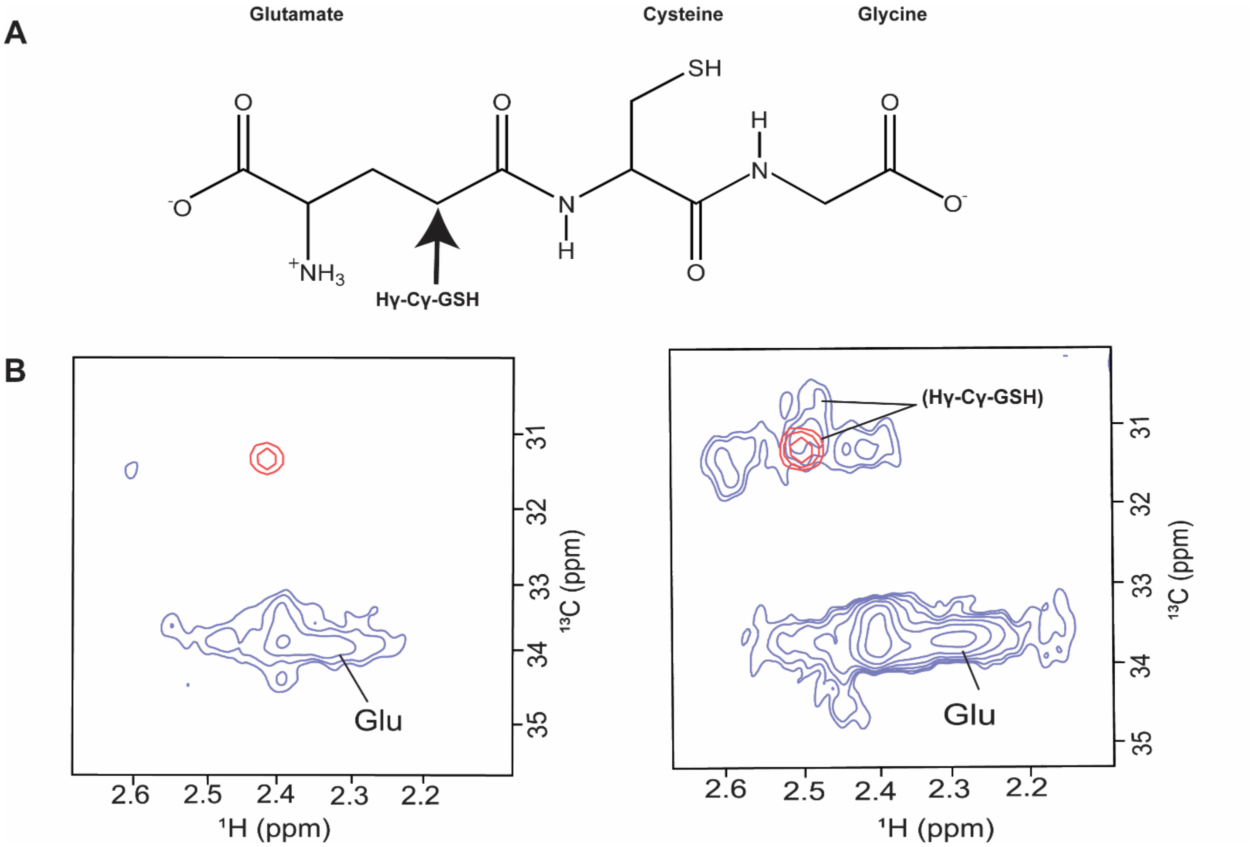

Figure S6. NMR assignments for glutathione and glutamate from Figure 3E. A) Chemical structure of glutathione, GSH, highlighting the H_g-C_g-GSH pair. B) ^1H-^{13}C HSQC spectra (blue) showing a glutamate peak and the H_γ-C_γ-GSH cross peak, which is absent in undifferentiated cells (left) and present in differentiated cells (right). The ^{13}C-^{13}C coupling is ≈60Hz. Cross peak assignment for H_γ-C_γ-GSH is in red.

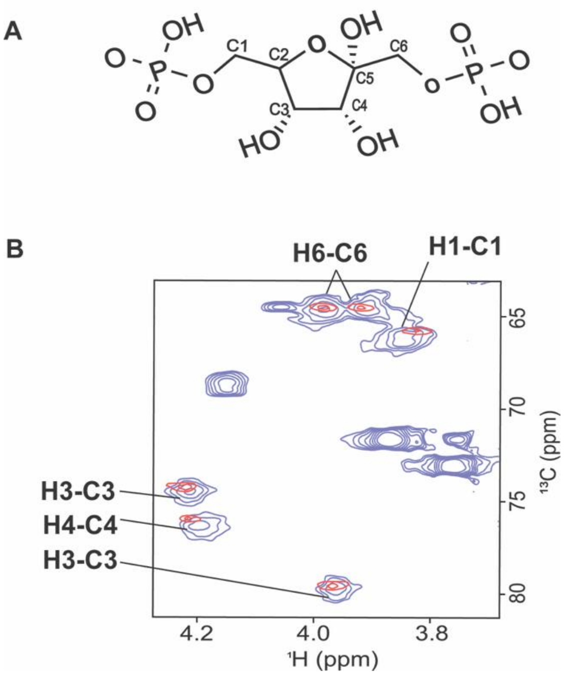

Figure S7. NMR assignments for fructose 1,6-biphosphate from Figure 3F. A) Chemical structure of fructose-6-biphosphate highlighting carbon atoms C1-C6. B) ^{1}H-^{13}C HSQC spectrum (blue) of FBP in undifferentiated cells with cross peak assignments (red). FBP was not detected in differentiated cells.

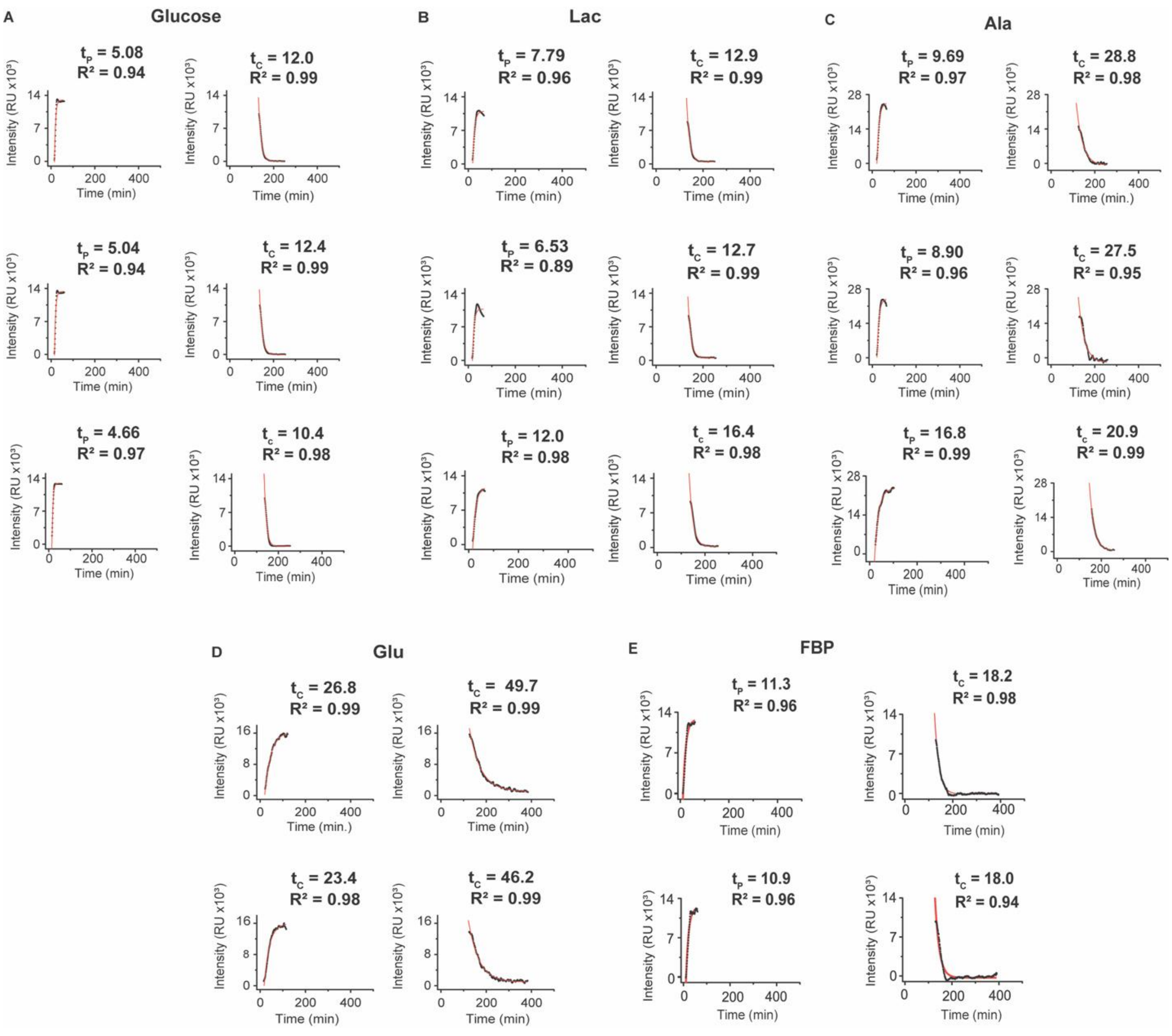

Figure S8. Kinetic flux analysis of undifferentiated SH-SY5Y cells. Single exponential fits to the leading and trailing edges of kinetic flux profiles yield production times (t_P) and clearance times (t_C) for metabolites. Data are in black and fits in red.

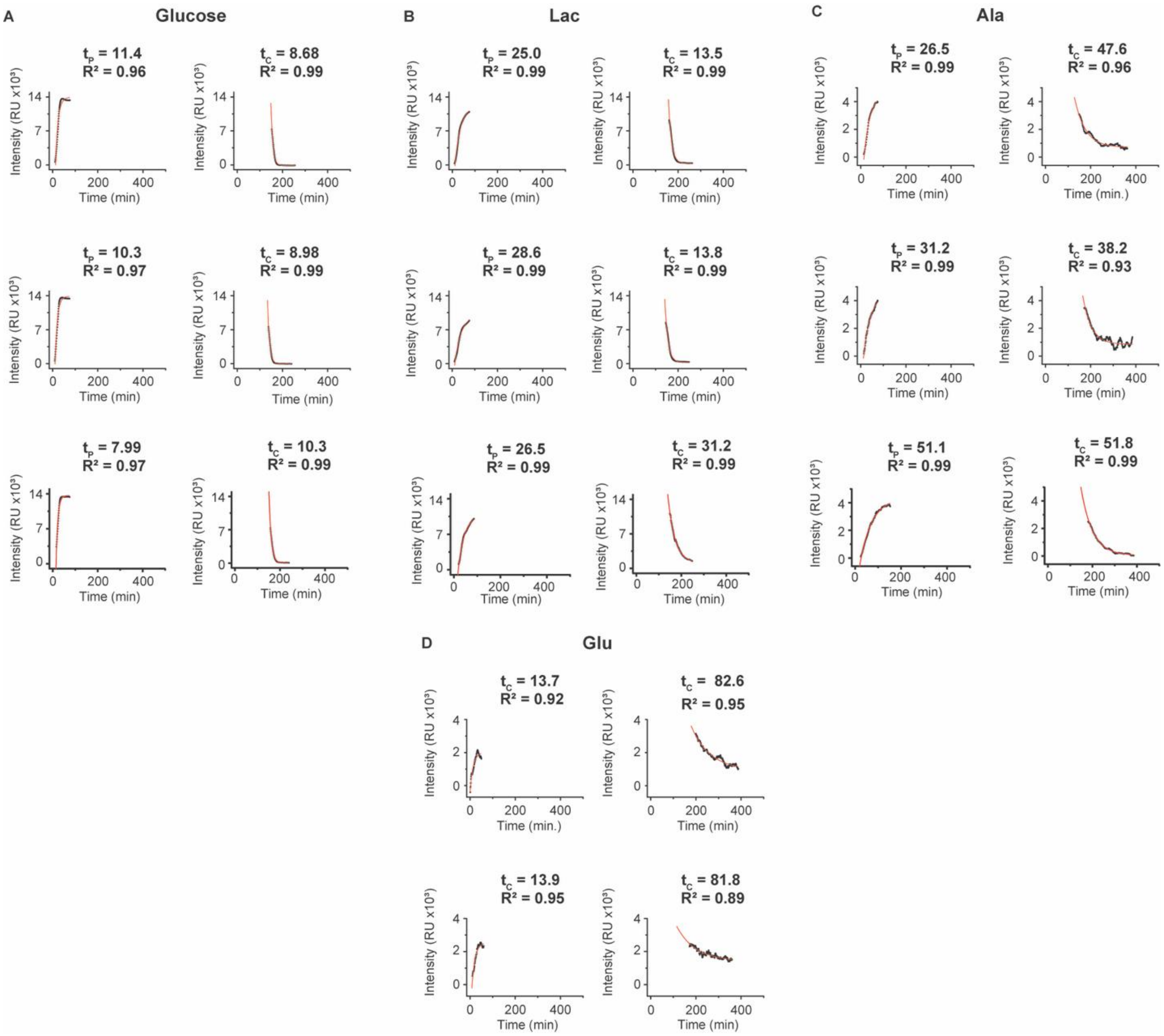

Figure S9. Kinetic flux analysis of differentiated SH-SY5Y cells. Single exponential fits to the leading and trailing edges of kinetic flux profiles yield production times (t_P) and clearance times (t_C) for metabolites. Data are in black and fits in red.

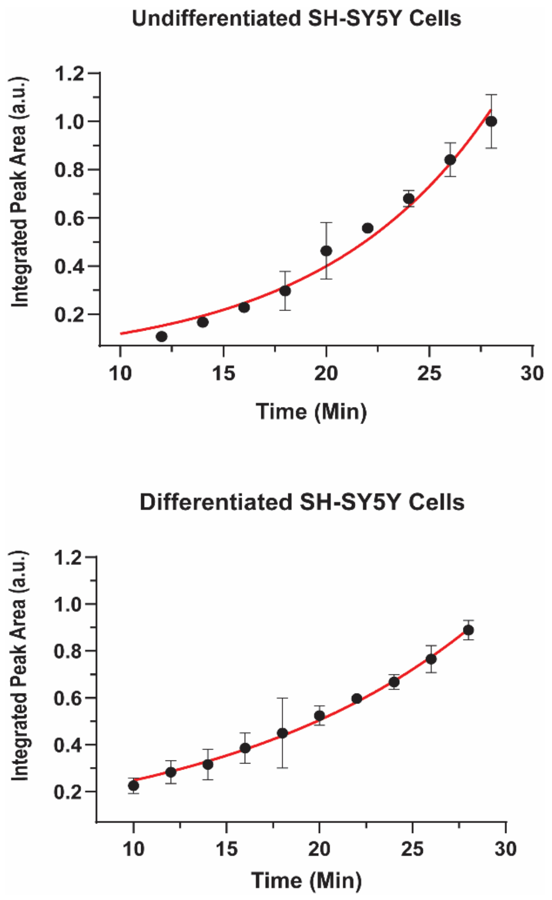

Figure S10. Time-dependent increase in lactate excretion. Integrated peak volumes of extracellular lactate for undifferentiated (top) and differentiated (bottom) SH-SY5Y cells. The data were fit using a nonlinear exponential growth model to evaluate the characteristic time of metabolite excretion, τ_{exc}. For differentiated cells τ_{exc} was 10 min and for undifferentiated cells τ_{exc} was 22 min. The intensities were normalized to the highest peak intensity.
